# Discovery and biochemical characterization of thermostable glycerol oxidases

**DOI:** 10.1007/s00253-023-12883-9

**Published:** 2024-01-06

**Authors:** Lars L. Santema, Laura Rotilio, Ruite Xiang, Gwen Tjallinks, Victor Guallar, Andrea Mattevi, Marco W. Fraaije

**Affiliations:** 1 Molecular Enzymology, University of Groningen, Nijenborgh 4, 9747AG, Groningen, The Netherlands; 2 Department of Biology and Biotechnology, University of Pavia, via Ferrata 9, 27100 Pavia, Italy; 3 Barcelona Supercomputing Center (BSC), Institució Catalana de Recerca i Estudis Avançats (ICREA), Barcelona, 08034 Spain

**Keywords:** Alditol oxidases, Flavin, Glycerol, Cell-free expression, In silico bioprospecting, Enzyme engineering

## Abstract

**Abstract:**

Alditol oxidases are promising tools for the biocatalytic oxidation of glycerol to more valuable chemicals. By integrating in silico bioprospecting with cell-free protein synthesis and activity screening, an effective pipeline was developed to rapidly identify enzymes that are active on glycerol. Three thermostable alditol oxidases from *Actinobacteria* Bacterium, *Streptomyces thermoviolaceus*, and *Thermostaphylospora chromogena* active on glycerol were discovered. The characterization of these three flavoenzymes demonstrated their glycerol oxidation activities, preference for alkaline conditions, and excellent thermostabilities with melting temperatures higher than 75 °C. Structural elucidation of the alditol oxidase from *Actinobacteria* Bacterium highlighted a constellation of side chains that engage the substrate through several hydrogen bonds, a histidine residue covalently bound to the FAD prosthetic group, and a tunnel leading to the active site. Upon computational simulations of substrate binding, a double mutant targeting a residue pair at the tunnel entrance was created and found to display an improved thermal stability and catalytic efficiency for glycerol oxidation. The hereby described alditol oxidases form a valuable panel of oxidative biocatalysts that can perform regioselective oxidation of glycerol and other polyols.

**Key points:**

*• Rapid pipeline designed to identify putative oxidases*

*• Biochemical and structural characterization of alditol oxidases*

*• Glycerol oxidation to more valuable derivatives*

**Graphical Abstract:**

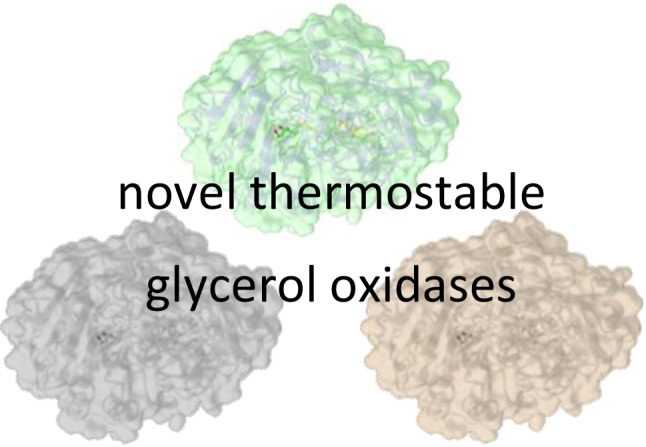

**Supplementary Information:**

The online version contains supplementary material available at 10.1007/s00253-023-12883-9.

## Introduction

The production of biodiesel generates overwhelming amounts of glycerol, up to 10% of the total yields (Abbaszaadeh et al. 2012). With its price constantly dropping, glycerol is slowly turning into a waste product, challenging the value of biodiesel as an alternative to fossil fuels (Morrison 2000; Quispe et al. 2013; Monteiro et al. 2018; Bagheri et al. 2015). Chemically, the oxidation of glycerol is generally afforded by means of metal catalysts, and the few available non-metal-based strategies require harsh conditions (Villa et al. 2015; Punniyamurthy et al. 2005; Gupta et al. 2017; Shi et al. 2019). Sustainable enzyme-based strategies for glycerol upcycling are thus highly sought after. Flavoenzyme oxidases are especially attractive for these purposes because they use molecular oxygen as electron acceptor, produce hydrogen peroxide as sole by-product, and give rise to valuable derivatives such as glyceraldehyde or glyceric acid (Bagheri et al. 2015; Ahmad et al. 2021; Wolfenden and Snider 2001; Mattevi 2006). Alcohol oxidases (EC 1.1.3.13) showed moderate activity on glycerol once their active site was engineered to become more wide and polar (Nguyen et al. 2018). Unfortunately, these enzymes are often purified with their flavin cofactor in the inactive semiquinone state, thus hampering their industrial use. On the other hand, certain alditol oxidases (AldO; EC 1.1.3.41) were found to be moderately active on glycerol without being affected by the inactivating semiquinone formation. However, until recently, the known AldOs suffered from either lack in thermostability, low activity, and/or poor expression levels (Heuts et al. 2007; Winter et al. 2012).

AldO from *Thermopolyspora flexuosa*, a thermophile, was recently described as a promising candidate for industrial glycerol oxidation, thanks to its high expression levels, good thermal stability, favorable kinetics properties, and efficient bioconversion of glycerol to glycerate (Chen et al. 2022). Prompted by this finding, we have performed a more extended analysis on different genomes, including those of thermophilic bacteria. We found that they harbor a vast number of putative AldOs with great potential as biocatalysts for glycerol oxidation. To rapidly explore and characterize this sequence trove, we set out to integrate computational modeling and experimental testing. In silico bioprospecting offers a cost-effective and efficient initial approach to increase the success rate of the discovery process. By first analyzing the activities of the putative enzymes computationally with the substrate of interest, we can eliminate non-active enzymes, reducing resource consumption (Kamble et al. 2019). Subsequently, high-throughput experimental methods can be applied to validate the simulation results in a synergistic manner. Multiple studies have shown that cell-free protein expression methods can nowadays allow efficient enzyme discovery because they can be directly coupled to an activity assay (Silverman et al. 2020; Garenne et al. 2021; Kwon and Jewett 2015; Tamiev et al. 2021; Rolf et al. 2019; Haslinger et al. 2021; Rolf et al. 2022). Moreover, they overcome the time-consuming constraints of working with cells and allow for rapid protein production bypassing most of the cloning, expression, and purification steps (Silverman et al. 2020). In this study, we harnessed this technology for identifying AldOs that are efficient in glycerol oxidation and feature properties that can make them applicable in industrial settings. Based on structural insights and using computational predictions, we designed a variant that successfully improves activity on glycerol. Our work expands the pool of biocatalysts that can be used for the selective oxidation of glycerol and other polyols and demonstrates the efficacy of cell-free protein expression for enzyme discovery.

## Materials and methods

### Chemicals and materials

The *E. coli* cell-free expression kit, NEBExpress® Cell-free *E. coli* Protein Synthesis System, and the restriction enzyme DPNI were bought from New England Biolabs. Chromatographic columns were from Cytiva. Other chemicals were acquired from Sigma-Aldrich.

### Genome mining

Using the sequence of AldO from *T. flexuosa* (GenBank: WP_142259226.1), the PSI-BLAST tool of the National Center for Biotechnology Information (NCBI) was utilized, using default settings, to search for homologues in thermophilic bacteria. Sequence alignment was done with ESpript (Robert and Gouet 2014). Numerous promising sequences were scrutinized, and their structures were predicated with the online AlphaFold tool ColabFold (Jumper et al. 2021; Mirdita et al. 2022). Homologues were selected based on the conservation of the active site residues (Heuts et al. 2007; Winter et al. 2012).

### Cloning, transformation, and mutagenesis

Using the Twist Biosciences tools, sequences encoding for the chosen candidate enzymes were codon optimized for expression in *E. coli* and BSAI sites were added to the 5’- and 3’-termini (Table S1). The synthetic genes were ordered from the same company and cloned via the Golden Gate methodology (Engler and Marillonnet 2014) in both His-SUMO-PET28a and pBAD His-SUMO vectors. A total of 2 μL of resulting PCR product was mixed with 40 μL CaCl_2_ competent NEB10β *E. coli* cells. After an incubation of 30 min on ice, the cells were heat shocked at 42 °C for 45 s and kept on ice again for another 5 min. The cells were allowed to recover in 250 μL LB-medium at 37 °C for an hour. Subsequently, 50 μL was plated on LB-agar plates containing 50 μg/mL ampicillin (amp) and incubated at 37 °C overnight. Cloning was verified with plasmid isolation and sequencing. Primers for mutagenesis (Table S2) were ordered from Eurofins Genomics, and mutagenesis was performed according to the QuickChange methodology (Kunkel 1985). The PCR mixture consisted of 12.5 μL PfuUltra II Hotstart PCR Master mix, 1 μM of both forward and reverse primer, 100 ng template plasmid, 2% DMSO, 0.8 μM MgCl_2_, and filled to a total volume of 25 μL with MilliQ water.

### Cell-free expression screening assay

Cell-free expression was performed with NEBExpress® Cell-free *E. coli* Protein Synthesis System. The protocol given by the supplier was followed, using 250 ng AldO homologue His_6_-SUMO-PET28a plasmid. 1.5 mL Eppendorf tubes were used as reaction chambers, and the incubation was done at 37 °C, 300 rpms for 4 h. A total of 10 μL of cell-free mixture was used to perform a horseradish peroxide (HRP) 4-aminoantipyrine (AAP)/3,5-dichloro-2-hydroxybenzenesulfonic acid (DCHBS) assay (Vojinović et al. 2004) together with 100 mM glycerol as substrate while using 50 mM KP_i_ pH 7.5 as reaction buffer. Product formation was followed with a synergy H1 microplate reader at 515 nm for 15 min at a constant temperature of 25 °C. A total of 2 μL of cell-free mixture was ran on a 12% sodium dodecyl sulfate-polyacrylamide gel electrophoresis (SDS-PAGE) for confirmation of protein expression.

### Protein expression, purification, and characterization

For the expression, overnight cultures were made in 5 mL LB supplemented with 50 μg/mL amp and incubated at 37 °C, 135 rpm. Each overnight culture was resuspended in 500 mL Terrific Broth medium containing 50 μg/mL amp, and the cultures were grown at 37 °C, 135 rpm in a non-baffled flask until an OD_600_ of ~0.6 was reached. Induction was performed with L-arabinose (0.02% final concentration), and cultures were incubated at 24 °C, 135 rpm for ~16 h. Cells were harvested by centrifuging (6000 rpm, 15 min, 4 °C), and pellets were stored at −20 °C.

For purification, cell pellets were resuspended into 50 mL lysis buffer (150 mM NaCl, 50 mM KP_i_, pH 8.0) and disrupted by sonication (5 s on 7 s off, 70% amplitude for 15 min). The supernatants were harvested by centrifuging at 11,000 rpm for 50 min at 4 °C and loaded onto Ni Sepharose gravity columns containing 2 mL lysis buffer. Columns were washed with 3 column volumes of wash buffer (50 mM KP_i_, 150 mM NaCl, 20 mM imidazole, pH 8.0), and the proteins were eluted of the columns using 3.5 mL of elution buffer (50 mM KP_i_, 150 mM NaCl, 500 mM imidazole, pH 8.0). Using PD10 columns, the elution buffer was exchanged for 50 mM KP_i_ pH 7.5, which was used as a storage solution.

Protein concentrations were determined using their flavin absorbance extinction coefficients, which were determined by flavin absorption comparison of enzymes before and after denaturation with 0.1% SDS, for 1 h at 25 °C. The unfolding temperatures of the enzymes were determined using the ThermoFAD method (Forneris et al. 2009). Enzymes were concentrated to ~200 μM in 50 mM KP_i_ pH 7.5 and diluted 20-fold in different buffers with a pH range from 5.0 to 9.0. The assay was preformed from 20 °C till 95 °C with 1 °C/30 s steps and monitored using a RT-PCR thermocycler (CFX96, Bio-Rad).

### Steady-state kinetics

Reactions were performed with 1.0 μM of enzyme and kept in 50 mM KP_i_ (pH 7.5) at 25 °C while stirring at 60 rpm in a 1 mL reaction chamber. Initial rates with glycerol were measured by following the consumption of oxygen at different substrate concentrations. The assay is convenient in that it directly detects co-substrate consumption by means of an oxygen electrode (Oxygraph plus, Hansatech Instruments Ltd.). For reference, also the kinetics on xylitol were determined using the established HRP-AAP/DCHBS assay (Vojinović et al. 2004) (JASCO V-660 spectrophotometer, ε_515_ = 26 mM^–1^ cm^–1^). Data were processed using GraphPad Prism 6.05 (La Jolla, CA, USA). Enzymatic activities at different pH values were analyzed at 25 °C with 1.0 μM of enzyme in 50 mM KP_i_, pH 7.5, and 500 mM of glycerol.

### Crystallographic studies

For the crystallization experiments, the purification protocol was slightly changed to optimize the sample purity. The cell-free extract was filtered with 0.45 μm filters (Merk) and loaded on a 5 ml Nickel column, previously equilibrated with lysis buffer (50 mM Hepes, 150 mM NaCl, 20 mM imidazole pH 8.0), using an Akta System (Cytiva) equipped with a multiwavelength detector (set at 280/350/450 nm). After loading was completed, the column was washed with lysis buffer until the absorbance at 280 nm returned to baseline levels. To elute bound proteins, a linear gradient of imidazole (20–500 mM) was applied to the column with an elution buffer consisting of 50 mM Hepes pH 8; 150 mM NaCl; and 500 mM imidazole. Fractions containing His-SUMO-AldO_*Ab*_ were pooled together and concentrated with Amikon 10k to a suitable volume. The resulting sample was incubated overnight with (1:300) homemade His-SUMO protease (1 mg/mL). While incubating, the protein mix was dialyzed overnight against 50 mM Hepes, pH 8; and 50 mM NaCl (storage buffer) to remove the excess of imidazole. The sample was then loaded on a His-Trap column (5 mL, Cytiva) to remove the His-tagged SUMO, while AldO_*Ab*_ was collected in the flow-through. The sample was gel filtered with a Superdex 200 10/300 column, previously equilibrated with storage buffer, and then concentrated to 20 mg/ml with Amikon (Merk) 10k. Vapor-diffusion sitting-drop crystallization was performed using the Oryx 8 robot (Douglas instrument) and different commercial kits at 20°C. AldO_*Ab*_ gave good diffracting crystals in conditions where monosaccharides were present as additives in the reservoir solution. Promising conditions were optimized by manually prepared sitting drop plates (Cryschem, Hampton) using 1+1 μL volumes. The crystals used for structure solution grew in the condition 0.12 M D-xylulose, 0.1 M HEPES and MOPS (acid) pH 7.5; 20% v/v PEG 500 MME; 10% w/v PEG 20’000. X-ray diffraction data were measured at 100 K on the PXI beamline of the Swiss Light Source in Villigen (SLS), Switzerland. Data were scaled using the XDS program (Kabsch 2010), and the structure was solved by molecular replacement (PhaserMR) (McCoy et al. 2007) using the respective AlphaFold (Jumper et al. 2021) coordinates as a search model. Iterative cycles of manual building and crystallographic refinement were performed with COOT and REFMAC5 (Murshudov et al. 2011) from the CCP4 suite (Emsley and Cowtan 2004). Figures were prepared with ChimeraX version 1.3 (Pettersen et al. 2004), and structural superposition was performed with DALI (Holm 2019).

### Protein preparation for in silico analysis

The models were generated using AlphaFold2 with the default options for the monomers, and the maximum template date was set to 2022-01-01. The per-residue confidence score (pLDDT) of the top-ranked models (rank_0.pdb) was validated, and the models were prepared using the protein preparation workflow in Schrodinger (Sastry et al. 2013). This workflow incorporates various functionalities, including protonation at a specified pH (in this case, pH 7.5, which corresponds to the pH of the enzymatic assays) and restrained minimization with a maximum root mean square deviation (rmsd) of 0.30 Å. Next, the substrate glycerol was docked using Glide (Friesner et al. 2006). A grid was generated for each protein, with the catalytic lysine as the center, following the default dimensions. The docking procedure was performed using standard precision, and three poses were extracted. The docking results were visually inspected, and the best docking pose, characterized by optimal distances between the catalytic atoms and the presence of correct hydrogen bonds, was selected for subsequent simulations.

### Protein Energy Landscape Exploration (PELE) simulations

PELE (Borrelli et al. 2005), a Monte Carlo-based sampling technique, was used to study protein-ligand interactions. In each step, random translations and rotations were applied to perturb the ligand. Additionally, the flexibility of the protein was considered by applying normal modes derived from the Anisotropic Network Model (ANM). Next, the side chains of the residues near the ligand are optimized with a library of rotamers to avoid steric clashes. Finally, a truncated Newton minimization is performed, and the new conformation is accepted or rejected according to the Metropolis criterion.

In all PELE simulations, the perturbation of the ligand was confined within a spherical box with a radius of 6 Å centered around the active site. The side chain optimization phase involved all residues within 6 Å of the ligand. The PELE simulations were executed on the MareNostrum IV cluster at the Barcelona Supercomputing Center (BSC) using 70 cores, with each core performing 1000 PELE steps.

The key variables analyzed and compared in these simulations were the enzyme-substrate interaction energies and the distances between the NZ atom of the “catalytic” lysine and the proton of the hydroxyl group of glycerol (specifically, both hydroxyl groups at the ends of the molecule). These parameters were calculated automatically at each step of the PELE simulations.

## Results

### Identification of thermostable alditol oxidase homologs

A PSI-BLAST was performed with the protein sequence of AldO_*Tf*_ from *T. flexuosa* (Chen et al. 2022) (GenBank: WP_142259226.1) as query input, targeting the genomes of known thermophilic bacteria and the NCBI’s non-redundant database; sequences of putative homologs were aligned using ESpript (Robert and Gouet 2014) (Fig. S1). One candidate, AldO_*St*_ from *Streptomyces thermoviolaceus* (GenBank: WP_189427891.1), was selected as the closest sequence in thermophilic bacteria. We chose three additional candidates from the NCBI’s non-redundant database based on sequence identity as well, namely AldO_*Ab*_ from *Actinobacteria* Bacterium (GenBank: PZN37415.1), AldO_*Msp*_ from *Microbispora sp. H10830* (GenBank: WP_220505403.1), and AldO_*Ch*_ from *Thermostaphylospora chromogena* (GenBank: WP_093262023.1). All four candidates shared greater than 60% sequence identities to AldO_*Tf*_.

**Fig. 1:**
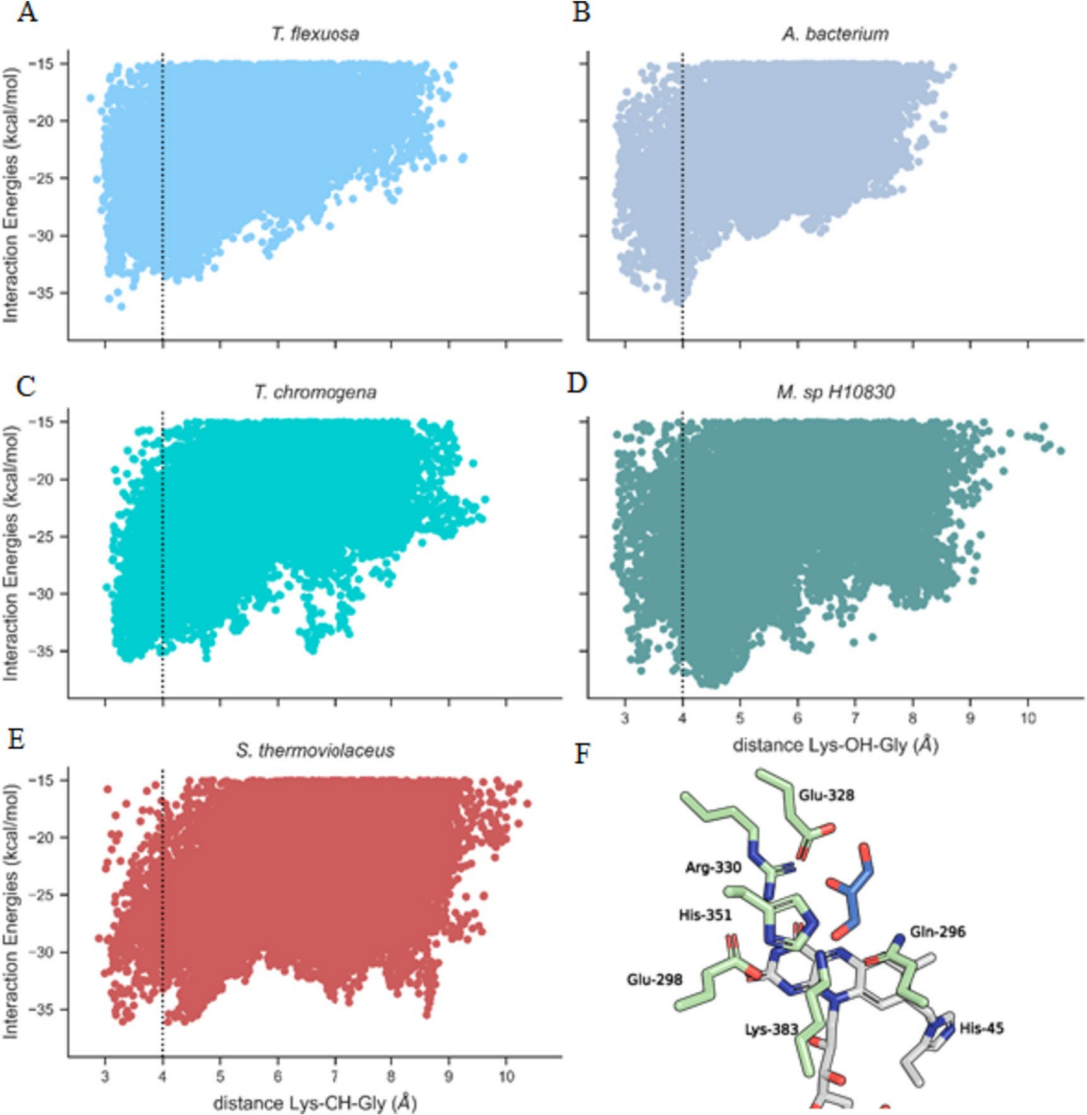
PELE simulation results using glycerol as the substrate ligand. The X-axis represents the distance in angstroms (Å) between the catalytic lysine residue and the hydroxyl group of the substrate. The Y-axis represents the interaction energies measured in kilocalories per mole (kcal/mol). The panels are labeled as follows: **A** AldO_*Tf*_ from *T. flexuosa*, **B** AldO_*Ab*_ from *A.* Bacterium, **C** AldO_Ch_ from *T. chromogena*, **D** AldO_*Msp*_ from *M. sp. H10830*. **E** AldO_*St*_ from *S. thermoviolaceus*. **F** The location of the active site residues with respect to glycerol in the predicted structure of AldO_*Tf*_

To evaluate the potential glycerol activities of these enzymes, their structures were predicted using AlphaFold2 (Jumper et al. 2021), and enzyme-substrate (glycerol) simulations were then carried out with our Monte Carlo software, PELE (Acebes et al. 2016) (Fig. 1A–E). AldO_*Tf*_ was included in the analysis for comparison. We considered the distance between the glycerol hydroxyl groups and the Nε atom of Lys383 (*T. flexuosa* residue numbering; Fig. S1), a conserved side chain that assists the deprotonation of the terminal O^1^ atom of the polyol substrate (Forneris et al. 2007) (Fig. 1F). The distances were plotted against the enzyme-substrate interaction energies. The location of the energetic minima in the scatterplots predicts higher enzymatic activities when the distance is shorter. Taking into account the symmetrical nature of glycerol, both its terminal hydroxyl groups were evaluated at each step and the one displaying the closest distance to the catalytic lysine was used in the plots. We applied a 4 Å cut-off, which represents a reasonably permissive range for proton transfer. Based on this criterion, Aldo_*Ch*_, Aldo_*Ab*_, and AldO_*St*_ were retained for further studies (Fig. 1A–C, E), whereas AldO_*Msp*_ was abandoned because the simulations revealed energy minima at distances well beyond the cut-off (Fig. 1D). It is important to note that the selection criteria were not overly strict as we aimed to have a more diverse pool of sequences.

### Cell-free screening of putative alditol oxidases

**Fig. 2:**
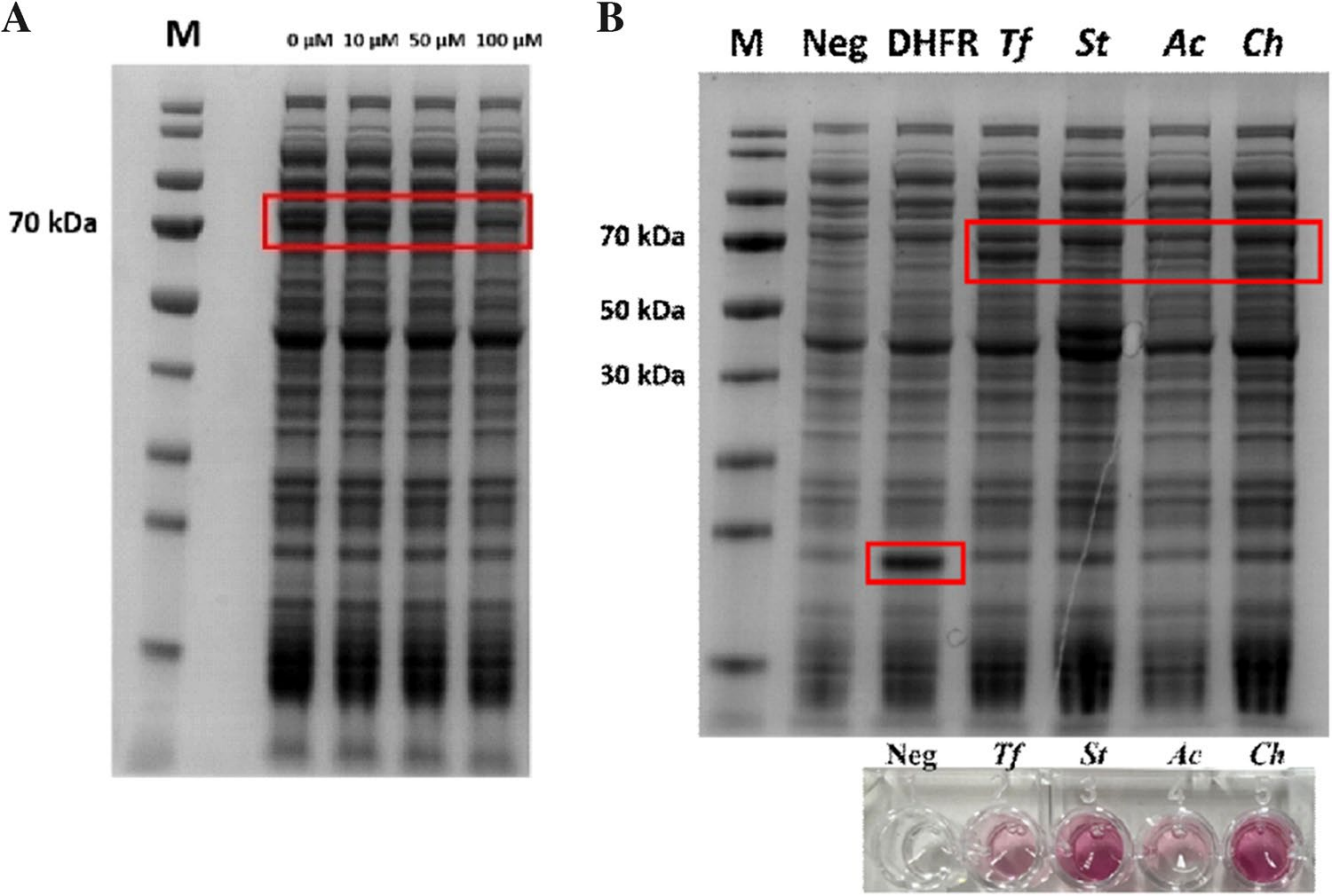
Optimization of cell-free protein expression. **A** Expression of 5-hydroxymethylfurfural oxidase (70 kDa) using the NEBExpress® Cell-free *E. coli* Protein Synthesis System. Expression was performed for 4 h at 37 °C and with different FAD concentrations (0–100 μM) and analyzed by SDS-PAGE (M: molecular weight markers). **B** SDS-PAGE analysis of cell-free expression of targeted oxidases. *Neg*, cell-free expression with no target gene; *DHFR*, positive control provided by New England Biolabs with a molecular weight of ~20 kDa; *Tf*, His-SUMO-AldO_*Tf*_ (~69 kDa); *St*, His-SUMO-AldO_*St*_ (~64 kDa); *Ac*, His-SUMO-AldO_*Ab*_ (~65 kDa); *Ch*, His-SUMO-AldO_*Ch*_ (~65 kDa). Expected positions are indicated with a red box. The inset shows the observed oxidase activity when the cell-free mixtures were tested with 100 mM glycerol using the HRP-AAP/DCHBS oxidase assay

As part of our strategy for rapid enzyme discovery, we explored cell-free protein synthesis for rapid expression and testing of novel oxidases. One of the most crucial factors influencing the protein yield of cell-free expression is the stability of the mRNA within the mixture (Ahn et al. 2005). By introducing a stem-loop structure, like a T7 promoter at the 3’-termini, the yield of the cell-free mixture can improve by at least a twofold (Ahn et al. 2005). We therefore chose the NEB Express® cell-free *E. coli* Protein Synthesis System that is based on a T7 RNA polymerase and thus requires a T7 promoter. In the literature, it is often described that different enzymes require different synthesis times and temperatures for their optimal synthesis (Rolf et al. 2019; Haslinger et al. 2021; Rolf et al. 2022). Our in-house experience showed that flavin-containing oxidases are well expressed in 1.5 mL Eppendorf tubes at 37 °C, while shaking at 800 rpm for 4 h. Longer synthesis times often lead to denaturation of enzymes. We also tested the effect of adding external FAD (up to 100 μM) to the cell-free system, but we did not observe a beneficial effect of flavin cofactor addition on expression levels (Fig. 2A). Using the optimized protocol, cell-free protein expression worked very well for all three selected flavoprotein oxidases that were expressed as His_6_-SUMO fusion proteins (Fig. 2B). For the initial screening, 10 μL of cell-free mixtures were tested using the HRP-AAP/DCHBS assay (Vojinović et al. 2004) together and 100 mM glycerol as substrate. Hydrogen peroxide formation was monitored at 515 nm with a synergy H1 microplate reader. The NEBExpress® cell-free mixture showed no background oxidase activity, and the activity of expressed AldO_*St*_, AldO_*Ab*_, and AldO_*Ch*_ was confirmed using AldO_*Tf*_ as a positive control (Fig. 2B). Thus, by coupling cell-free synthesis to a relatively facile HRP-based oxidase assay, a direct cloning and screening pipeline was developed for identifying glycerol oxidase. As the oxidase assay is generic, the same protocol can be used for any other hydrogen peroxide generating oxidase.

### Expression and spectral characterization of the thermostable alditol oxidases

For further biochemical characterization, larger amounts of purified enzymes were required compared to the yields of the cell-free expression system. Our work also included the previously discovered AldO_*Tf*_ used as a benchmark (Chen et al. 2022). The AldO-encoding genes were cloned into a pBAD vector with an N-terminal His_6_-SUMO tag. The enzymes were recombinantly overexpressed in *E. coli* NEB10-β, and high protein yields were obtained upon affinity purification: AldO_*Tf*_, 300 mg protein/L culture; AldO_*Ab*_, 72 mg/L; AldO_*Ch*_, 30 mg/L; AldO_*St*_, 71 mg/L. Such levels are significantly higher when compared with that previously obtained for AldO from *Acidothermus cellulolyticus* 11B (2.5 mg/L) (Winter et al. 2012). After incubation in 5% v/v acetic acid, the SDS-PAGE separated proteins displayed bright fluorescence under UV light confirming the presence of a covalently bound flavin (Fraaije et al. 1997) (Fig. S2). Consistently, the enzyme absorbance spectra exhibited two maxima at around 350 and 450 nm, with shoulders at 425 and 475 nm. The two absorption maxima are typical for an oxidized flavin cofactor, and the relatively low wavelength of the maximum at 350 nm is an emblematic characteristic of a histidyl-bound 8α-substituted flavin (Jong et al. 1992; Singer and Edmondson 1980). By SDS denaturation and using FAD as reference, the extinction coefficients of the bound FAD at 452 nm for all AldOs could be determined: 12.5 mM^−1^ cm^−1^ for AldO_*Tf*_, 12.2 mM^−1^ cm^−1^ for AldO_*Ab*_, 12.3 mM^−1^ cm^−1^ for AldO_*Ch*_, and 15.7 mM^−1^ cm^−1^ for AldO_*St*_.

### Stability of the alditol oxidases

**Fig. 3:**
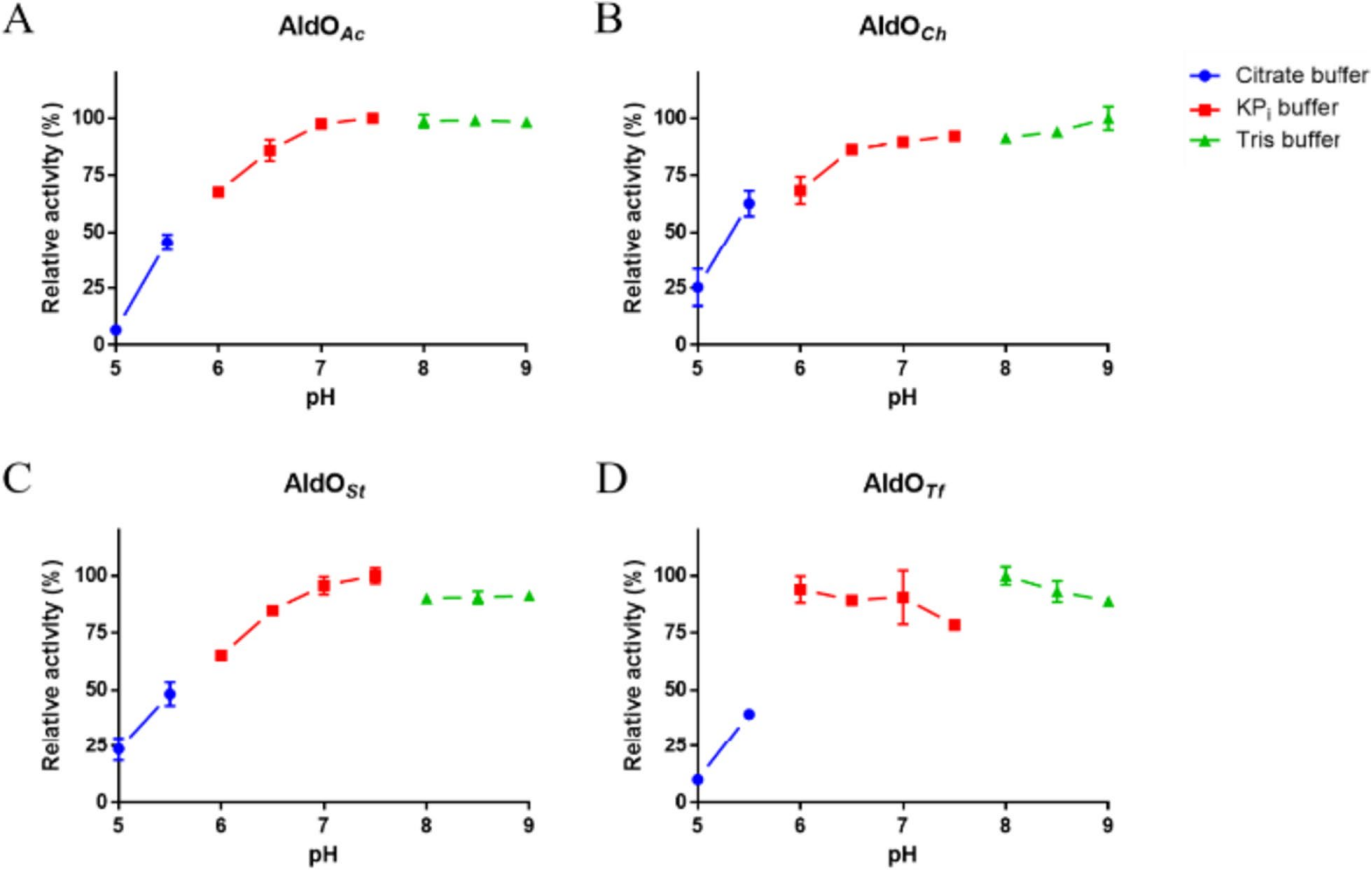
pH dependence of AldO activity. **A** AldO_*Ab*_. **B** AldO_*Ch*_. **C** AldO_*St*_. **D** AldO_*Tf*_. 100 mM glycerol was used to measure oxidase activity

All three newly identified AldOs are favorably endowed with good thermostability properties, and their melting temperatures are as large as 85 °C at pH values higher than 5.5–6.0 (Table 1). Similar pH-dependent profiles were observed also for their enzymatic activities (Fig. 3). AldO_*St*_ emerged as the most thermostable enzyme and, together with AldO_*Ch*_, the only one that featured some residual activity at acidic pH values. These properties are comparable or better than those exhibited by AldO_*Tf*_ (Chen et al. 2022). Remarkably, all investigated AldOs can be stored at 4 °C without any activity loss even after months.

**Table 1:** Thermal stabilities at different pH values. The melting temperatures of the reported alditol oxidases were measured in different buffers (50 mM)

Condition	Melting temperature T_m_ (°C)				
	AldO_*Ab*_	AldO_*Ch*_	AldO_*St*_	AldO_*Tf*_	V258L_P259I AldO_*Tf*_
Citrate buffer pH 5.0	68	62	70	61	65
Citrate buffer pH 5.5	77	73	80	71	75
KP_i_ pH 6.0	80	75	82	72	77
KP_i_ pH 6.5	80	76	84	73	79
KP_i_ pH 7.0	82	77	85	75	81
KP_i_ pH 7.5	82	78	85	76	81
Tris-HCl pH 8.0	81	77	84	76	80
Tris-HCl pH 8.5	82	77	84	76	80
Tris-HCl pH 9.0	81	76	84	75	80

### Steady-state kinetics

Steady-state kinetic parameters were determined for both xylitol and glycerol as substrates (Table 2; Fig. S3). In contrast with previous literature, AldO_*Tf*_ (Chen et al. 2022) proved more active with xylitol than glycerol. Hence, it does not break the trend shown by the known AldOs which favor xylitol over glycerol. All investigated AldOs showed a decrease in their rates of catalysis when the hydrogens on glycerol are replaced by deuterium. This observation suggests that the reaction rate is limited for a large part by the glycerol oxidation step involving a hydride transfer from the substrate to the flavin (Table S3) (Belleau and Moran 2006). Remarkably, besides AldO_*St*_, the newly reported AldOs in this study are all superior glycerol oxidases as compared to the previously described AldOs from *A. cellulolyticus* and *Streptomyces coelicolor*, especially regarding the K_M_ values (Heuts et al. 2007; Winter et al. 2012) (Table 2). In this regard, it is noticeable that AldO_*St*_ showed a non-catalytic second minimum in the PELE simulations, providing a rationalize for its higher K_M_ value and corroborating our strategy for enzyme selection based on the catalytic distance cut-off (Fig. 2D, E). The *k*_cat_ of 6373.1 s^−1^ for AldO_*Tf*_ on glycerol reported in Chen et al. (2022) is much higher than all *k*_cat_ values that we determined for all four investigated AldOs, including the enzyme from *T. flexuosa* (1.6–4.2 s^−1^; Table 2). To our knowledge, *k*_cat_ values greater than 100 s^−1^ have rarely been reported for flavoprotein oxidases. Besides this discrepancy with the previous literature, AldO_*Tf*_ showed the highest catalytic efficiency, while AldO_*St*_ exhibited the highest *k*_cat_ value. Combined with their good thermal stability, the kinetics and stability data outline these two enzymes as the most valuable candidates for industrial usage (Tables 1 and 2).

**Table 2:** Steady-state kinetic parameters. The steady-state kinetic parameters of the reported alditol oxidases were determined

	Organism of origin	*K*_*M*_ (mM)		*k*_*cat*_ (s^−1^)		*k*_*cat*_*/ K*_*M*_ (M^−1^ s^−1)^		References
		Xylitol^a^	Glycerol^b^	Xylitol^a^	Glycerol^b^	Xylitol	Glycerol	
AldO_*Sc*_	*S. coelicolor*	0.32	350	13	1.6	4.1 × 10^4^	4.6	Heuts et al. 2007
AldO_*Ac*_	*A. cellulolyticus*	0.07	270	1.9	1.3	2.7 × 10^4^	4.8	Winter et al. 2012
AldO_*Ab*_	*A.* Bacterium	0.03	184	4.2	2.6	14 × 10^4^	14	this study
AldO_*Ch*_	*T. chromogena*	0.04	143	3.5	2.0	12 × 10^4^	14	this study
AldO_*St*_	*S. thermoviolaceus*	0.02	523	1.9	4.2	9.5 × 10^4^	8	this study
AldO_*Tf*_	*T. flexuosa*	0.03	50	3.1	1.6	10 × 10^4^	32	this study
V258L_P259I AldO_*Tf*_	*T. flexuosa*	0.04	41	4.3	4.0	11 × 10^4^	98	this study
V257L_P258I AldO_*Ab*_	*A.* Bacterium	n.d.	157	n.d.	1.4	n.d.	9	this study

^a^The HRP-coupled assay was used for measuring kinetics

^b^Oxygen consumption was measured for determining the kinetic parameters

### Structural elucidation of AldO_Ab_ in complex with D-xylulose

**Fig. 4:**
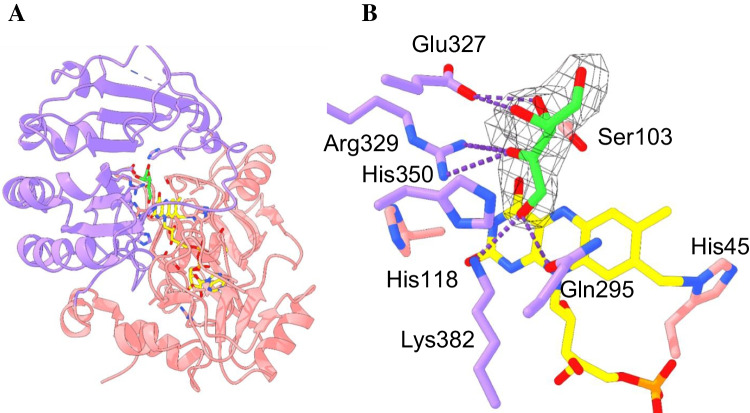
The crystal structure of AldO_Ab_ in complex with D-xylulose. **A** The FAD (residues 1–179/387–426) and substrate (180–386) domains are in pink and purple, respectively. Residues 228–239 that belong to a flexible loop were not modelled into the electron density and are represented as a dashed line. **B** Close-up view of the active-site bound D-xylulose. Several side chains are engaged in H-bond interactions with the ligand (dashed lines). The carbon atoms of FAD and D-xylulose are colored in gold and lime, respectively. Oxygen atoms are shown in red and nitrogen atoms in blue. The unbiased electron density 2F_o_-F_c_ of D-xylulose is contoured at 1.5 σ level

The three discovered AldOs were tested for crystallization. AldO_*Ch*_ did not crystallize, while AldO_*St*_ crystals were affected by merohedral twinning and were not further pursued for structural studies. AldO_*Ab*_ was crystallized with xylulose, the product of xylitol oxidation. The crystals diffracted to 2.4 Å resolution, yielding electron density maps of excellent quality (Table 3).AldO_*Ab*_ is structurally very similar to the homologous enzyme from *S. coelicolor* (PDB entry 2VFR (Forneris et al. 2007)) with a root-mean-square deviation of 1.0 Å for 404 aligned Cα atoms and 61% sequence identity. The overall structural assembly is composed by 14 α helices and 14 β sheets that are organized into two distinct domains, as observed in other members of the VAO family (Mattevi et al. 1997): an FAD-binding domain and a substrate-binding domain (Fig. 4A). The planar flavin cofactor sits at the domain interface with its 8α-methyl group covalently bound to the Nδ1 of His45. The presence of this covalent linkage is a characteristic feature of the VAO-like enzymes although the bond typically involves the Nε2 rather than the Nδ1 atom of the flavinylated histidine side chain (Leferink et al. 2008). The flavin cofactor further interacts with the protein through many H-bonds that are mostly mediated by the residues belonging to the loop 43-47 and the side chain of Ser103 that H-bonds to the flavin N5. This network of interactions maintains the flavin buried within the protein scaffold. The residues that compose the active site wrap the isoalloxazine on its *re* side (Fig. 4B). They include Gln295, Glu327, Arg329, His350, and Lys382 belonging to the substrate binding domain, and Ser103, His118, and His45 being part of the FAD binding domain. The conformation of these side chains is highly conserved when compared to the active site arrangement observed in AldO from *S. coelicolor.* A well-ordered molecule of D-xylulose, present in the reservoir solution of the crystallization condition, was clearly visible in the electron density (Fig. 4B). The ligand is buried in the catalytic pocket, and its OH groups are engaged in H-bonds with the surrounding side chains. His350 and Lys382 are located at the bottom of the substrate site where they interact with the C1-O1 group of the D-xylulose bound in front of the reactive N5 atom of the flavin. This geometry is perfectly suited for the hydride transfer from the substrate C1 carbon leading to aldehyde or carboxylic (e.g., glycerate from glycerol) products as observed in the AldO enzymes so-far characterized (Heuts et al. 2007; Winter et al. 2012; Chen et al. 2022).

**Table 3.** Crystallographic statistics

AldO_*Ab*_ in complex with D-xylulose (8OT8)	
Resolution range	49.5–2.4 (2.5–2.4)
Space group	P2_1_2_1_2_1_
Unit cell axes (Å)	56.44 109.91 296.71
Total reflections^a^	488355 (48012)
Unique reflections^a^	73280 (7224)
Multiplicity^a^	6.7 (6.6)
Completeness^a^ (%)	99.70 (99.82)
Mean I/sigma(I) ^a^	8.44 (1.01)
Wilson B-factor	49.77
R-merge^a, b^	0.1649 (2.169)
CC1/2^a,c^	0.998 (0.649)
Reflections used in refinement^a^	73273 (7224)
R-work^a^	0.2463 (0.4752)
R-free^a^	0.2886 (0.4573)
N. of non-hydrogen atoms	12710
Macromolecules	12414
Ligands	252
Solvent	44
Protein residues^d^	1627
RMS (bonds) (Å)	0.015
RMS (angles) (°)	1.89
Ramachandran favored (%)	92.08
Ramachandran allowed (%)	7.42
Ramachandran outliers (%)	0.50

^*a*^Values in parentheses are for reflections in the highest resolution shell

^*b*^R_merge_=Σ|*I*_*i*_-<*I*>|/Σ*I*_*i*_, where I_i_ is the intensity of *i*^th^ observation and <*I*> is the mean intensity of the reflection

^c^The resolution cut-off was set to CC_1/2_^,^> 0.3 where CC_1/2_ is the Pearson correlation coefficient of two “half” data sets, each derived by averaging half of the observations for a given reflection

^d^The asymmetric unit contains four protein chains

**Fig. 5:**
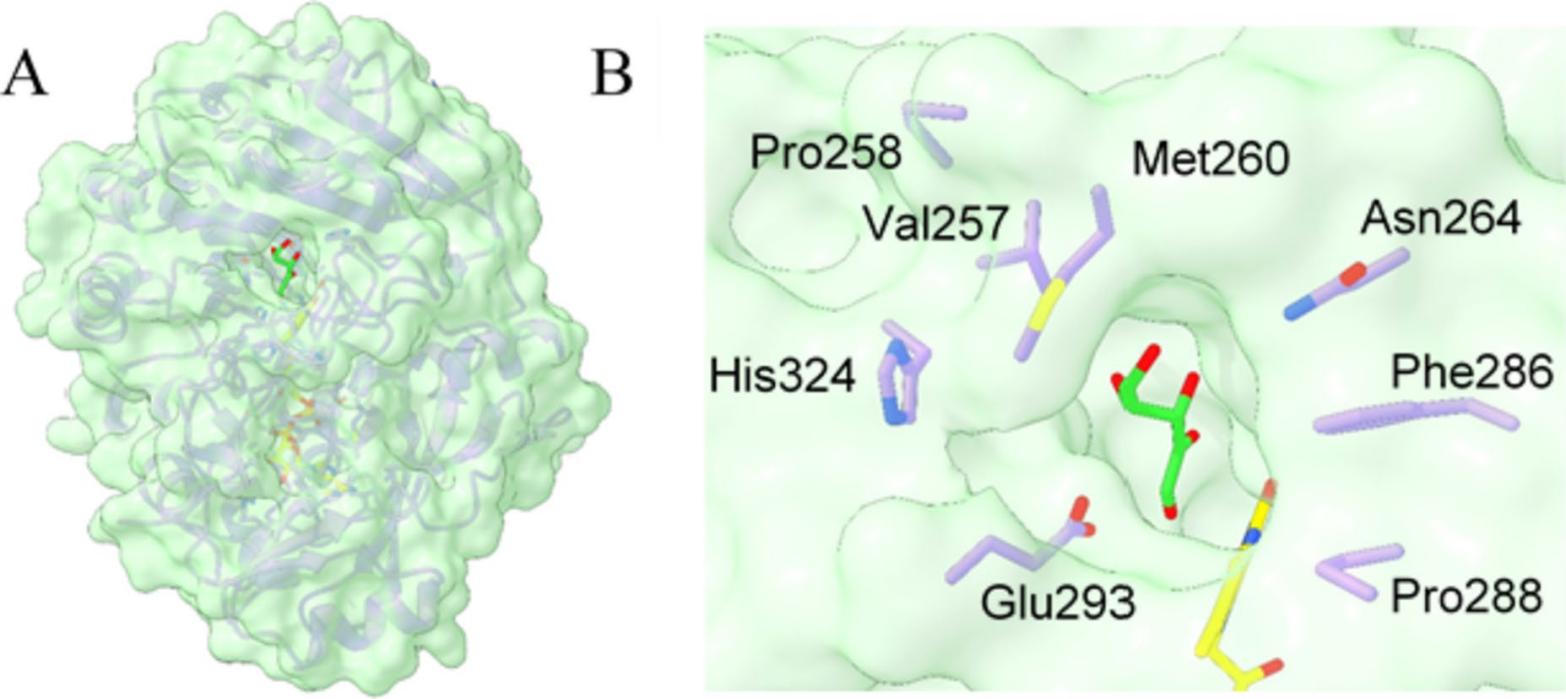
The tunnel leading to the active site in AldO_Ab_. **A** Semitransparent molecular surface showing the entrance of the tunnel that leads to the catalytic pocket. **B** Close-up of the side chains that describe and define the rim of the tunnel entrance. The carbon atoms of FAD and D-xylulose are colored in gold and lime, respectively. Oxygen atoms are shown in red and nitrogen atoms in blue

When inspecting the molecular surface of AldO_*Ab*_, a noticeable feature is the presence of a wide tunnel that connects the active site to the external surface (Fig. 5A). A set of highly conserved side chains (Fig. S1) circumscribes the tunnel entrance: Val257, Pro258, Met260, Asn264, Phe286, Pro288, Glu293 and His324 (Fig. 5B). It is conceivable that the substrate may initially loosely interact with these residues. It can then gain access to the flavin through the tunnel passageway. With a diameter of about 10 Å and a length of 14 Å, the tunnel is long and wide enough to bind polyols of different lengths and sizes (Table 2**)**. Remarkably, the side chains forming both the tunnel wall and the active site display ordered conformations as judged from their well-defined electron densities. Glycerol, the focus of our work, can be assumed to bind in the same position and conformation of the C1-C2-C3 skeleton of the D-xylulose ligand without the imposing any large conformation changes on the surrounding protein residues.

### Improved mutant design by substrate induced-fit simulations

**Fig. 6:**
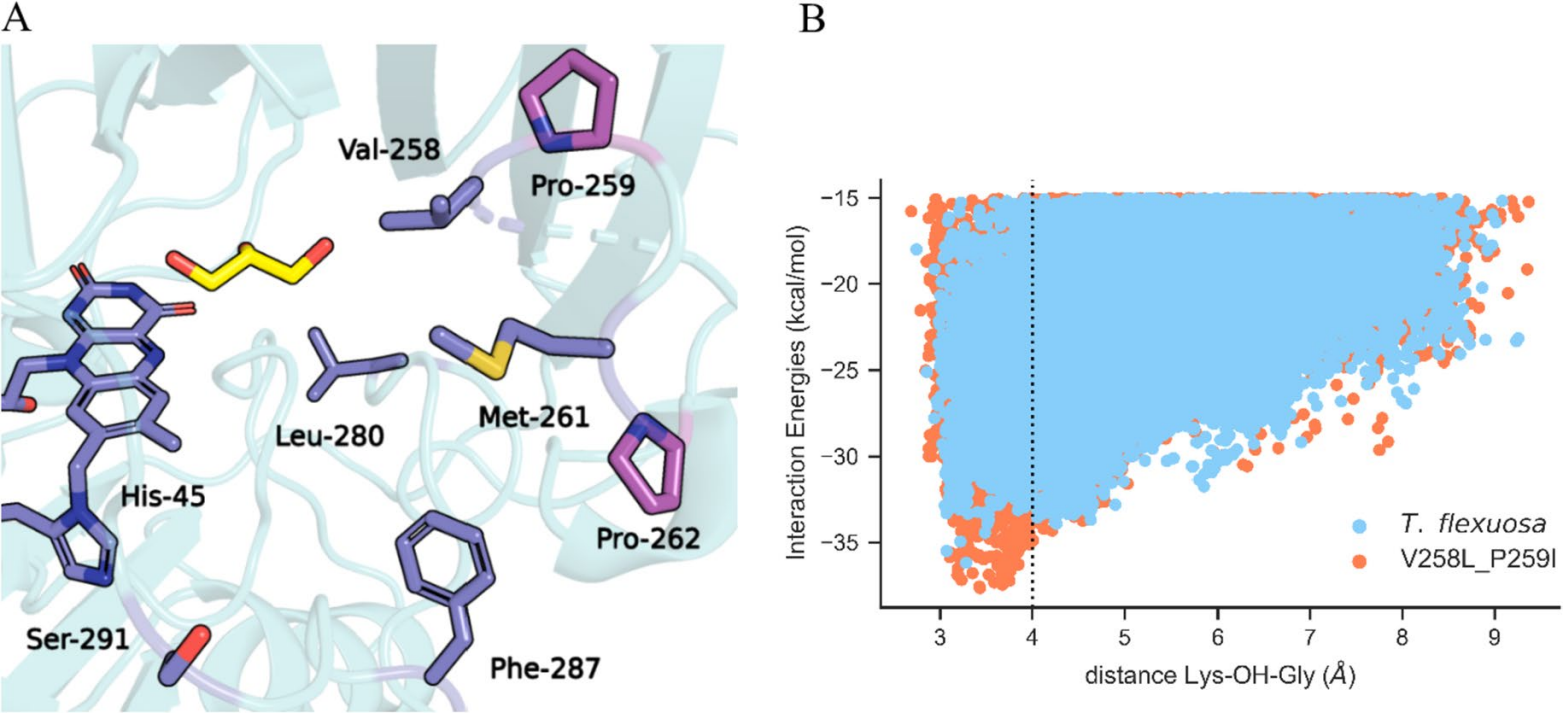
AldO_Tf_ engineering. **A** In purple are the positions detected by Hotspot Wizard for the AlphaFold structure of AldO_*Tf*_ and in pink the prolines that were selected for mutagenesis because they were next to Val258 and Met261. **B** Scatter plot of the simulation results for the double mutant V258L_P259I and comparison with the data from the wild-type enzyme. Val258 and Met261 are homologous to Val257 and Met260 of AldO_*Ab*_ (Fig. S1)

Based on the steady-state kinetic analysis of the different AldOs (Table 2), AldO_*Tf*_ showed the highest enzymatic efficiency and was then selected for the subsequent step of in silico engineering. It is widely recognized that most protein mutations lead to destabilization, resulting in a trade-off between activity and stability when attempting to optimize catalytic properties (Teufl et al. 2022). To mitigate the consequences of altering naturally evolved sequences, various strategies can be employed. One such strategy involves phylogenetic analysis to identify evolutionary variable regions that are more tolerant to mutations. This functionality is implemented in HotSpot Wizard (Sumbalova et al. 2018) which ranks the different amino acid positions according to the evolutionary variability. HotSpot Wizard identified five mutable positions that stood out because they are located around in the entrance of the catalytic tunnel as predicted from the AldO_*Ab*_ crystal structure and the AldO_*Tf*_ AlphaFold model: Val258, Met261, Leu280, Phe287, and Ser291 (Figs. 5B and 6A) Among these positions, Val258 and Met261, which were closest to the ligand, were selected for in silico mutagenesis. Moreover, the next contiguous residues Pro259 and Pro262, respectively, were also targeted for mutagenesis to construct double mutants. Combining contiguous residues seems to increase the probability of cooperative effects between them (Reetz and Carballeira 2007). In total, we analyzed 50 single and double mutants using PELE (Table S4). The eligible mutations for each site were limited to those present in a multiple sequence alignment constructed using Consurf (Yariv et al. 2023). Since we aimed at a clear increase in activity, the selection criteria in this phase were more stringent compared to one used in bioprospecting: whether the energetic minimum is located at a similar or better catalytic distance and exhibits improved interaction energies compared to the wild type. Based on this criteria, eight variants were selected for experimental validation (Table S4), with the double mutant V258L_P259I showing a three-fold increase in the *k*_*cat*_*/*K_M_ value mostly due to an improvement in the *k*_*cat*_ (Table 2). PELE’s simulations for this double mutant convincingly showed an improvement in the catalytic local minimum with a similar catalytic distance but better interaction energies (−37.65 kcal/mol compared to −36.19 kcal/mol of the wild type) and a significant higher density of points as compared to the wild-type simulation (Fig. 6B). Interestingly, the activity towards xylitol also seems to be improved in the same fashion, and coincidently, the double mutant appeared to improve the thermostability of the enzyme by 5 °C (Table 1).

The reasons for the improved catalytic performance afforded by the double mutation are difficult to fully rationalize. They reflect a combination of subtle structural and dynamic effects on the substrate diffusion and fine positioning in the active site. The subtlety of these phenomena can be appreciated by considering that introducing the same double mutation in AldO_*Ab*_ did not deliver a significant improvement in the catalytic parameters despite the very high 88% sequence identity between the two proteins (Table 2; data shown in Fig. S3). Therefore, transferring mutations between highly identical sequences is not as straightforward as it may seem reflecting the multitude of factors contributing to enzymatic activities.

## Discussion

Our work focused on extending the biocatalytic toolbox of oxidases acting on glycerol. The first glycerol oxidase was reported in 1980 (Uwajima et al. 1980). The enzyme was purified from a fungus, *Aspergillus japonicus* AT008, and found to be a multimeric heme- and copper-containing enzyme (Uwajima et al. 1984). Unfortunately, no sequence information on this reported glycerol oxidase is available, and no specific activity was reported. In 2007, we identified a bacterial alditol oxidase from *S. coelicolor* (AldO_*Sc*_) which, except efficiently oxidizing typical alditols such as xylitol and sorbitol, was also active on glycerol (Heuts et al. 2007) (Table 4). Interestingly, the oxidase was found to catalyze a double oxidation of polyols, yielding the corresponding acids as products (van Hellemond et al. 2009). A thermostable variant, AldO_*Sc*_, was discovered in 2012 displaying very similar catalytic properties (Winter et al. 2012). Using directed evolution, it has been attempted to improve the catalytic efficiency of AldO_*Sc*_ on glycerol (Gerstenbruch et al. 2012; Zhang et al. 2021). This let to limited success with the best variant (an 11-fold mutant) displaying only a 2-fold improvement in *k*_cat_/K_M_ (Table 4). In 2014, another fungal oxidase was reported to be active on glycerol (Linke et al. 2014). We have studied this alcohol oxidase from *Phanerochaete chrysosporium* (PcAOX) in more detail and could only detect a very low activity on glycerol (Nguyen et al. 2018). Yet, through elucidation of its crystal structure and subsequence structure-inspired engineering, a variant (F101S mutation) was created that improved its efficiency on glycerol by 50 fold (Table 4). Recently, another homolog of AldO_*Sc*_ was reported to display an extremely high activity on glycerol: AldO_Tf_ (*k*_cat_ of 6373.1 s^−1^) (Chen et al. 2022). Such high activity is in stark contrast with that observed for the other known AldOs (Table 4). Intrigued by this finding, we explored this newly reported AldO and several other homologs. In our hands, AldO_Tf_ was found to display a similar *k*_cat_ when compared with other AldOs (Table 4). Yet, its K_M_ value is relatively low, rendering it one of the most effective glycerol oxidases known. By computational-assisted enzyme engineering, we managed to improve its kinetic properties towards glycerol. The V258L_P259I double mutant of AldO_Tf_ displays the lowest K_M_ value and highest *k*_cat_ known for a glycerol (Table 4). Except for a good catalytic efficiency, the engineered variant is also highly thermostable. Such an engineered alditol oxidase is an attractive biocatalyst for producing D-glyceric acid, while it may also be considered for biosensing purposes.

**Table 4:** Biocatalytic properties of reported glycerol oxidases

Organism of origin	Enzyme	*k*_cat_ (s^−1^)	K_M_ (mM)	*k*_cat_/K_M_ (s^−1^mM^−1^)	T_M_ (°C)	References
*S. coelicolor*	AldO_*Sc*_	1.6	350	4.6	61	Heuts et al. 2007
*A. cellulolyticus 11B*	AldO_*Ac*_	1.3	270	4.8	84	Winter et al. 2012
*P. chrysosporium*	PcAOX	-	-	0.1	58	Nguyen et al. 2018
*P. chrysosporium*	PcAOX *F101S mutant*	3.0	580	5.0	51	Nguyen et al. 2018
*S. coelicolor*	AldO_*Sc*_ *11-fold mutant*	0.57	67	8.5	n.d.	Chen et al. 2022
*T. flexuosa*	AldO_*Tf*_	1.6	50	32	76	This study
*T. flexuosa*	AldO_*Tf*_ *2-fold mutant*	4.0	41	98	81	This study

To expand the enzymatic toolbox for glycerol oxidation, a pipeline that involved a two-step process was established: initial selection of enzyme candidates from a sequence database through molecular simulations, followed by coupling cell-free expression to an oxidase assay. Within a relatively short time span compared to traditional methods, this protocol allowed us to identify three alditol oxidases endowed with higher activity on glycerol than previously described AldOs. A similar approach was recently shown to be successful for another class of flavoenzymes, FMN-containing azoreductases (Rolf et al. 2022). The enzymes targeted in this study contain FAD as a prosthetic group. The observation that the cell-free expression resulted in functional FAD-containing oxidases confirms that such an expression approach is compatible with enzymes that need to incorporate a flavin cofactor.

The three newly discovered alditol oxidases reported in this study proved to be highly thermostable, prefer alkaline conditions, and were obtained in relatively high yields of 30–70 milligrams of protein per liter of culture. We observed similarly convenient properties, including sustained glycerol oxidase activity, also for the previously discovered AldO_*Tf*_ from *T. flexuosa* (Chen et al. 2022). The structural elucidation of AldO_*Ab*_ from *Actinobacteria* Bacterium showed a closed tunnel leading to the flavin. This active site architecture is suited for the selective recognition of polyols of different lengths as outlined by the binding of a xylulose present in the crystal structure. Using the structural information, substrate-induced-fit simulations were performed that identified a conserved Val-Pro pair located at the tunnel entrance as hotspot to improve the activity towards glycerol. While mutating these residues in AldO_*Ab*_ afforded little improvement, mutating AldO_*Tf*_ resulted in a three-fold increased activity towards glycerol combined with improved thermostability by 5 °C. Collectively, these data highlight how in silico bioprospecting, cell-free protein expression, combined with robust protocols for biochemical, structural, and computational analysis can rapidly deliver promising candidates for oxidative bioconversions. The hereby described AldOs represent state-of-the-art enzymes for the conversion of glycerol and other polyols.

## Supplementary information

ESM 1 (PDF 611 kb)

## Data Availability

All data generated and analyzed in this study is included in this published article and its supplementary information document.
