# Supplementary material for: Discovery and biochemical characterization of thermostable glycerol oxidases
Source: Appl Microbiol Biotechnol. 2024 Jan 6;108:61. doi: 10.1007/s00253-023-12883-9 (PMC10771423; doi:10.1007/s00253-023-12883-9)
Supplement: Supplementary file 1 [file 253_2023_12883_MOESM1_ESM.pdf]

Supplementary information

Discovery by cell-free protein synthesis and biochemical characterization of thermostable glycerol oxidases

Lars L. Santema[a]#, Laura Rotilio[b]#, Ruite Xiang[c]#, Gwen Tjallinks[a],
Victor Guallar[c]*, Andrea Mattevi[b]* & Marco W. Fraaije[a]*

[a] Molecular Enzymology, University of Groningen, Nijenborgh 4, 9747AG Groningen, The Netherlands

[b] Department of Biology and Biotechnology, University of Pavia, via Ferrata 9, 27100 Pavia, Italy

[c] Electronic and atomic protein modelling group, Barcelona Supercomputing Center, E-08034 Barcelona, Spain.

# Equally contributed

* Corresponding authors: victor.guallar@bsc.es, andrea.mattevi@unipv.it, m.w.fraaije@rug.nl

This additional file contains:

Tables: S1, S2, S3, S4 (page 4 - 6)

Figures: S1, S2, S3 (page 2 & 3)

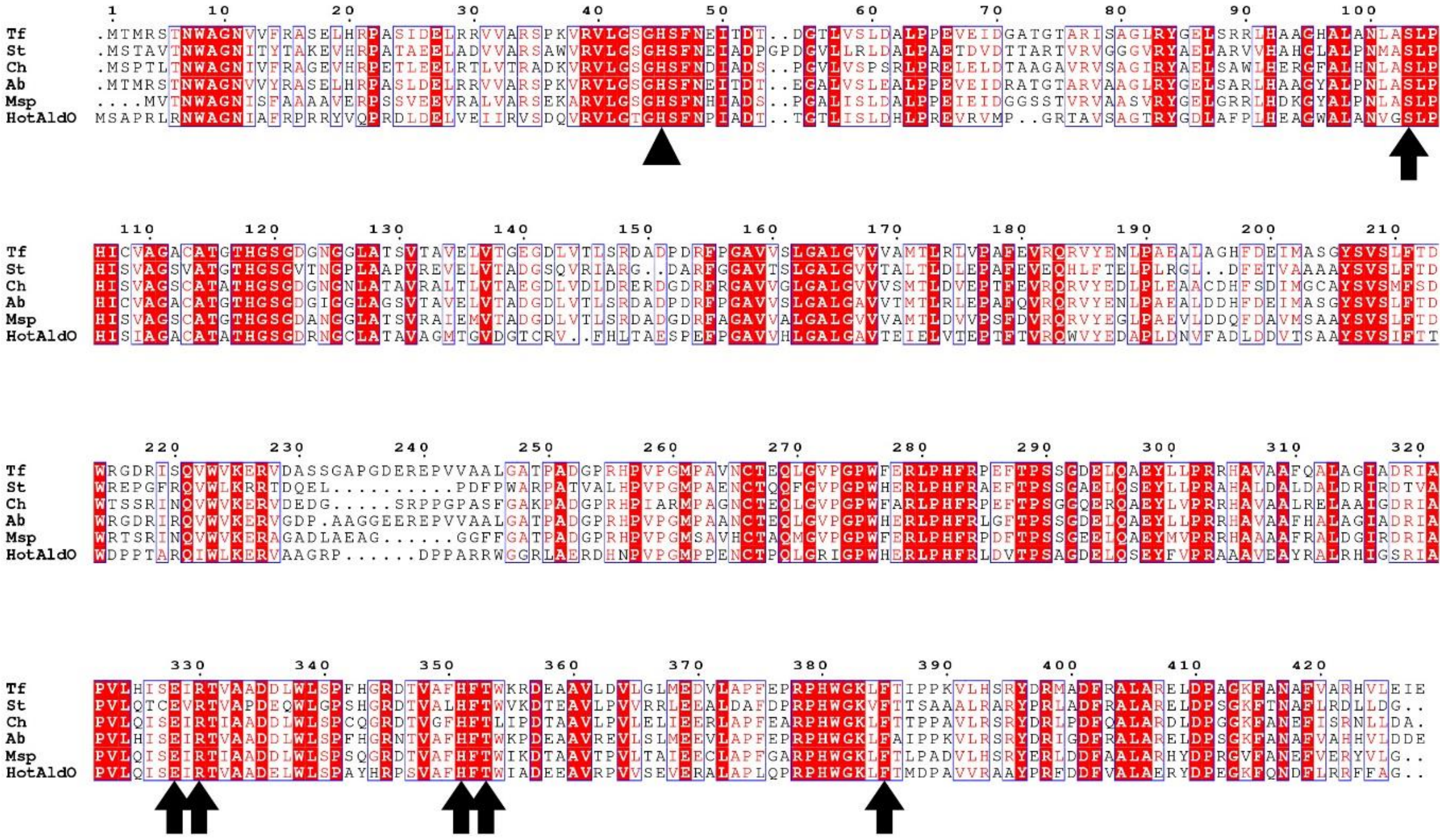

Figure S1. **Multiple sequences alignment of experimentally tested AldOs**. Conserved residues are shown with a red background. The triangle marks the conserved FAD-binding histidine and the arrows mark the conserved active-site residues. The aligned AldO sequences are: AldO$_{Tf}$ (Tf), AldO$_{St}$ (St) AldO$_{Ch}$ (Ch), AldO$_{Ab}$ (Ab) and AldO$_{Msp}$ (Msp). The previously reported AldO from *A. cellulolyticus* (HotAldO) is also shown for reference (Winter et al. 2012). Generated with ESpript (Robert and Gouet, 2014).

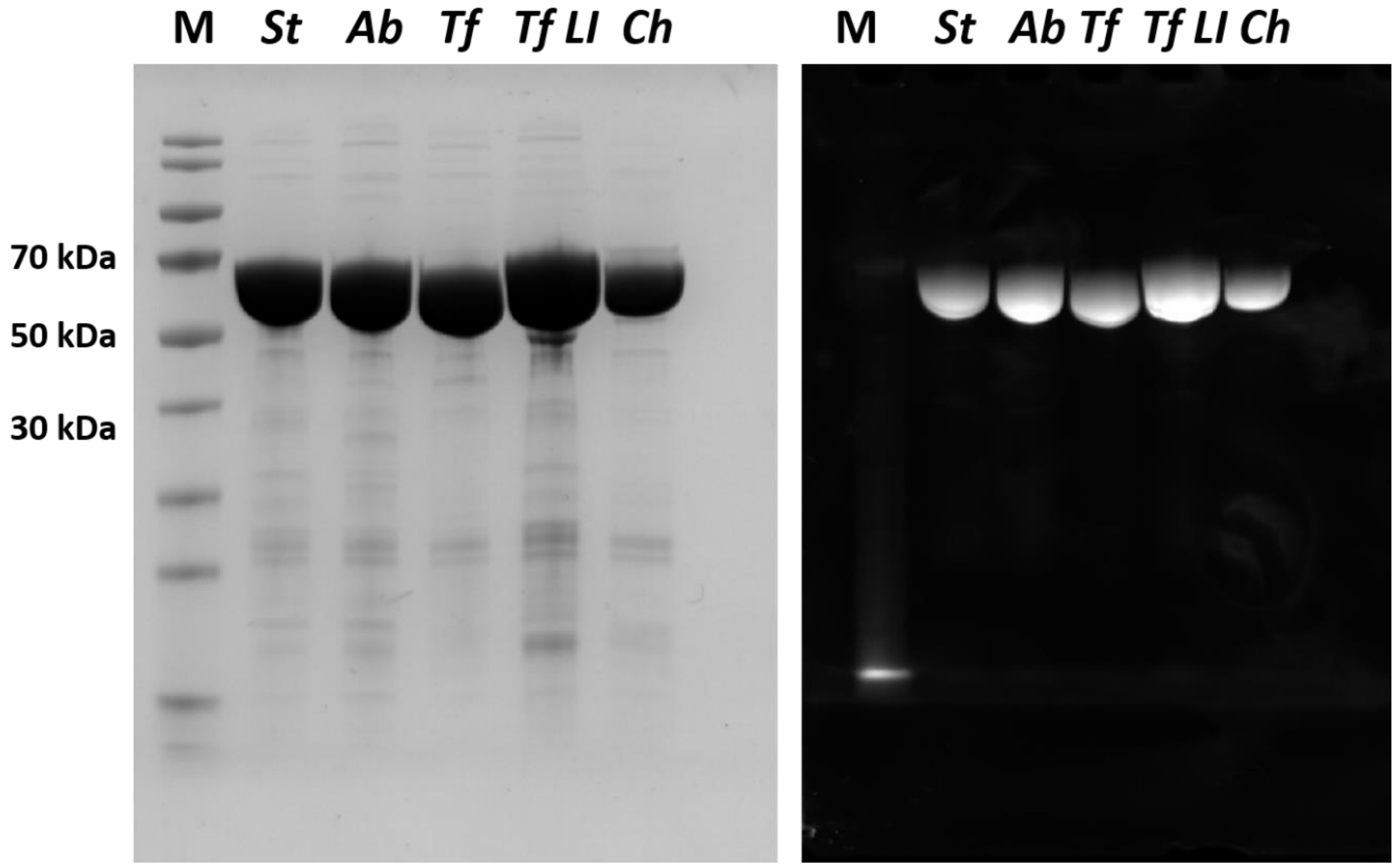

Figure S2. **Purification of the AldO enzymes. A:** SDS-PAGE analysis of purified His$_6$-SUMO tagged AldOs. The left image shows the Coomassie-stained gel whereas and the right panels are for acetic acid (5% v/v) staining under UV light. M: molecular weight markers; St: AldO$_{St}$; Ab: AldO$_{Ab}$; Tf: AldO$_{Tf}$; *Tf LI*: V258L_P259I AldO$_{Tf}$; Ch: AldO$_{Ch}$.

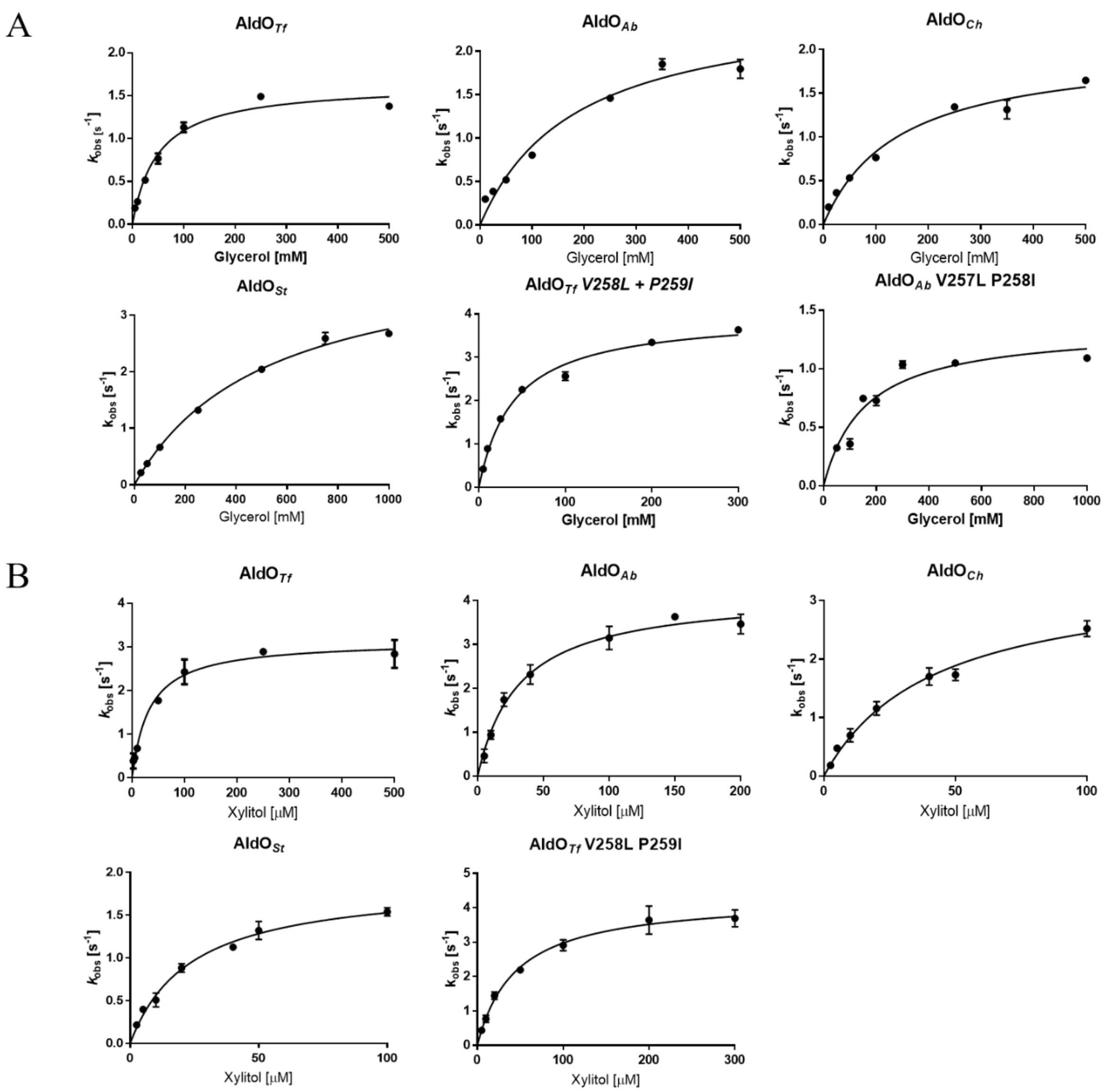

Figure S3. Michaelis-Menten kinetics for studied AldOs on glycerol (**A**) and xylitol (**B**).

***Table S1.* Sequences of *E.coli* codon optimized synthetic genes**

Name of gene	Sequence ‘3 – ‘5
AldO$_{Ab}$	ATGACAATGCGTTCGACAAACTGGGCGGGCAATGTAGTATACCGGGCTTCAGAGCTTCACCGTCCAGCATCACTTGACGAGC<br>TTAGACGTGTTGTCGCACGATCCCCTAAGGTTCGGGTCTTAGGTTCAGGTCACTCTTTCAATGAGATAACTGACACAGAGGG<br>AGCTTTGGTTAGTTTAGAGGCACTCCCGCCCGAGGTTGAGATCGACCGTGCCACAGGGACAGCAAGAGTCGCCGCAGGTTT<br>GCGGTACGGTGAGTTATCGGCTAGACTCCACGCAGCAGGGTACGCATTACCTAATTTGGCTTCACTTCCTCACATCTGTGTTG<br>CTGGGGCATGTGCCACAGGGACACACGGAAGTGGTGACGGAATCGGTGGATTGGCTGGGTCAGTTACTGCAGTCGAGCTTG<br>TTACAGCAGACGGTGACCTTGTAACGTTAAGTCGAGACGCCGACCCAGACCGTTTCCCTGGAGCCGTTGTTAGTTTAGGAGC<br>CCTTGGTGCCGTTGTAACGATGACTTTACGTCTTGAGCCTGCATTCCAAGTCCGGCAACGTGTCTACGAGAATTTACCAGCA<br>GAGGCACTCGACGACCACTTCGACGAGATCATGGCATCTGGTTACAGTGTATCACTTTTCACTGACTGGCGAGGAGATCGTA<br>TCCGTCAAGTTTGGGTCAAGGAGAGAGTTGGTGACCCTGCAGCTGGTGGTGAGGAGCGTGAGCCTGTTGTCGCTGCTCTCGG<br>TGCCACACCAGCCGACGGTCCTAGACATCCCGTTCCTGGGATGCCAGCAGCTAATTGTACTGAGCAATTGGGAGTACCAGGG<br>CCCTGGCACGAGCGTTTACCCCACTTCAGATTGGGTTTCACTCCCTCGTCAGGAGACGAGTTGCAAGCAGAGTACTTATTAC<br>CCAGAAGACACGCAGTTGCAGCCTTCCACGCTTTAGCAGGTATAGCTGACAGAATAGCACCCGTTCTTCACATATCCGAGAT<br>ACGTACGGTAGCAGCAGACGACTTGTGGTTATCGCCTTTCCACGGTCGTAATACTGTCGCATTCCACTTCACATGGAAGCCA<br>GACGAGGCCGCCGTTCGTGAGGTTTTATCACTCATGGAAGAGGTCTTGGCACCATTCGAGCCACGTCCTCACTGGGGCAAGT<br>TGTTCGCCATACCCCCTAAGGTTCTTCGAAGTAGATACGACCGTATAGGTGACTTCCGAGCACTTGCTAGAGAGTTAGACCC<br>CTCTGGGAAGTTCGCCAATGCCTTCGTTGCCCACCACGTTTTAGACGACGAGTAG
AldO$_{Ch}$	ATGTCGCCCACTTTAACGAATTGGGCCGGTAATATCGTATTCCGAGCTGGAGAGGTCCACCGTCCCGAGACACTTGAGGAGT<br>TGAGAACTTTGGTTACACGTGCAGACAAGGTCCGTGTACTTGGGTCCGGACACTCGTTCAATGACATCGCCGACAGTCCTGG<br>TGTCTTAGTCAGTCCTTCGCGTTTACCACGAGAGCTTGAGCTTGACACAGCTGCCGGGGCAGTACGAGTCTCAGCTGGTATC<br>AGATACGCTGAGCTTTCCGCATGGTTGCACGAGAGAGGATTCGCCCTTCACAATTTAGCATCATTGCCCCACATCTCTGTAG<br>CAGGAAGTTGTGCCACGGCAACGCACGGATCTGGGGACGGTAATGGGAATCTTGCTACGGCAGTTAGAGCCCTCACACTCG<br>TTACGGCTGAGGGAGACTTAGTCGACTTAGACCGTGAGAGAGACGGTGACCGATTCCGGGGAGCCGTTGTTGGTTTGGGTG<br>CATTAGGAGTCGTTGTTTCGATGACACTCGACGTTGAGCCAACATTCGAGGTCCGGCAAAGAGTCTACGAGGACCTTCCATT<br>GGAAGCAGCATGTGACCACTTCTCTGACATCATGGGGTGTGCCTACTCCGTAAGTATGTTCTCGGACTGGACATCATCCCGT<br>ATCAATCAAGTCTGGGTTAAAGAGCGAGTTGACGAGGACGGGAGTAGACCGCCTGGGCCTGCATCTTTCGGAGCAAAGCCT<br>GCCGACGGACCACGGCACCCCATCGCCAGAATGCCAGCTGGTAATTGTACTGAGCAATTAGGGGTTCCCGGGCCTTGGTTCG<br>CACGACTTCCACACTTCCGACCTGAGTTCACGCCCAGTGGTGGGCAAGAGCGTCAAGCCGAGTACTTAGTTCCTCGTCGTCA<br>CGCTGTTGCAGCACTCCGAGAGCTTGCCGCCATAGGTGACCGAATAGCCCCAGTATTACAAATATCTGAGATCCGTACAATC<br>GCCGCAGACGACCTCTGGTTATCACCATGTCAAGGGCGGGACACTGTTGGGTTCCACTTCACTCTTATACCCGACACAGCAG<br>CAGTACTCCCCGTTTTAGAGCTTATCGAGGAGCGGTTAGCACCCTTCGAGGCTAGACCACACTGGGGAAAGCTCTTCACTAC<br>ACCCCCAGCTGTTCTTCGTTCTCGTTACGACCGTTTACCCGACTTCCAAGCCCTTGCACGTGACCTCGACCCAGGTGGGAAGT<br>TCGCAAATGAGTTCATCTCGCGGAATCTCCTCGACGCTTGA
AldO$_{St}$	ATGTCGACAGCTGTAACTAATTGGGCCGGAAATATAACTTACACGGCAAAAGAGGTTCACCGTCCAGCAACAGCCGAGGAG<br>TTGGCAGACGTAGTAGCTCGAAGTGCCTGGGTTAGAGTTCTTGGATCAGGACACTCATTCAATGAGATAGCAGACCCAGGA<br>CCAGACGGTGTATTGCTTCGGCTTGACGCACTTCCAGCCGAGACTGACGTCGACACGACTGCACGGACAGTTCGTGTAGGCG<br>GCGGGGTACGATACGCCGAGCTTGCACGTGTTGTCCACGCACACGGTTTGGCCCTTCCAAATATGGCCTCGTTGCCTCACAT<br>CTCAGTCGCTGGATCAGTAGCCACGGGAACGCACGGTAGTGGGGTTACAAATGGTCCACTTGCAGCTCCCGTCCGAGAGGTT<br>GAGTTAGTCACGGCAGACGGGTCCCAAGTACGGATCGCCCGGGGTGACGCCAGATTCGGCGGTGCTGTTACGTCTCTTGGTG<br>CCTTAGGTGTAGTTACGGCCTTAACACTTGACCTCGAGCCTGCATTCGAGGTTGAGCAACACTTGTTCACAGAGTTACCATTG<br>CGTGGACTCGACTTCGAGACAGTTGCTGCTGCTGCATACTCCGTCTCGTTGTTCACTGACTGGCGGGAGCCAGGATTCCGGC<br>AAGTATGGCTCAAGCGTCGTACAGACCAAGAGCTTCCCGACTTCCCATGGGCACGACCTGCAACTGTTGCCCTTCACCCTGT<br>CCCTGGGATGCCAGCCGAGAATTGTACACAACAATTCGGAGTCCCCGGTCCTTGGCACGAGCGTCTTCCTCACTTCCGTGCA<br>GAGTTCACACCCTCGTCTGGAGCCGAGCTCCAATCTGAGTACTTATTACCCCGGGCACACGCCTTAGACGCTTTAGACGCCC<br>TTGACCGAATACGTGACACTGTAGCACCCGTTTTGCAAACTTGTGAGGTTCGAACAGTTGCACCTGACGAGCAATGGTTGGG<br>TCCTTCACACGGTCGTGACACGGTTGCTCTCCACTTCACGTGGGTAAAGGACACTGAGGCAGTACTTCCTGTCGTTCGTCGGT<br>TGGAAGAGGCATTAGACGCTTTCGACCCACGTCCACACTGGGGAAAGGTTTTCACAACGTCCGCAGCTGCATTGCGTGCCCG<br>GTACCCACGGTTGGCTGACTTCAGAGCACTTGCTCGGGAGCTTGACCCCTCAGGAAAGTTCACTAA
AldO$_{Tf}$	ATGACAATGCGTAGTACCAACTGGGCTGGCAACGTAGTATTCCGAGCGAGCGAGCTTCACCGCCCCGCGTCCATTGACGAGT<br>TACGGCGGGTGGTGGCAAGATCTCCCAAGGTACGAGTGCTGGGAAGTGGACACTCATTCAACGAGATTACTGACACGGACG<br>GTACTCTCGTGTCATTAGATGCTCTGCCACCCGAGGTTGAGATCGACGGCGCAACCGGTACAGCTCGAATTAGCGCGGGATT<br>ACGATATGGTGAACTGAGCCGACGTCTCCATGCCGCGGGGCATGCGTTAGCTAACCTGGCGAGCTTACCTCACATCTGCGTG<br>GCTGGGGCCTGCGCAACTGGGACACATGGCAGCGGTGATGGAAACGGTGGACTGGCTACCTCAGTGACCGCTGTCGAACTC

	GTTACAGGCGAAGGGGATCTGGTTACATTATCTCGTGACGCGGATCCAGATCGATTCCCCGGTGCGGTAGTGAGCCTTGGCG<br>CTTTAGGAGTCGTGGTCGCGATGACGCTCAGACTGGTGCCGGCTTTTGAAGTTCGCCAACGCGTATACGAAAACCTGCCGGC<br>AGAAGCGCTGGCTGGTCACTTCGACGAGATTATGGCCTCGGGGTACAGTGTGTCCCTGTTCACAGACTGGCGTGGCGACCGT<br>ATTTCACAGGTTTGGGTTAAAGAGCGTGTAGACGCATCATCTGGTGCACCGGGAGACGAGAGAGAGCCGGTGGTGGCTGCG<br>CTGGGTGCCACACCAGCAGATGGTCCTCGCCATCCTGTGCCTGGAATGCCTGCAGTGAATTGCACGGAACAACTTGGGGTTC<br>CAGGGCCTTGGTTTGAGCGGCTGCCTCACTTTCGGCCAGAGTTCACACCGTCGTCAGGCGACGAGCTGCAGGCCGAGTATTT<br>ATTACCACGCCGACACGCCGTCGCAGCGTTCCAAGCGCTCGCGGGAATAGCCGACCGTATCGCTCCGGTGTTACATATCTCT<br>GAAATCAGAACGGTGGCCGCAGACGACCTCTGGCTGAGTCCCTTCCACGGTAGAGATACTGTAGCATTCCATTTCACATGGA<br>AGCGGGACGAAGCCGCTGTTCTTGATGTCTTAGGTCTGATGGAAGACGTACTCGCCCCTTTCGAGCCGCGCCCTCACTGGGG<br>TAAGTTGTTCACGATACCACCGAAAGTTCTGCATTCCCGTTACGACCGCATGGCTGACTTTCGGGCTCTGGCGCGCGAATTG<br>GACCCGGCAGGGAAGTTCGCTAACGCCTTCGTTGCTCGCCATGTGCTGGAGATTGAATAA

Table S2. **Used primers for mutagenize**

Primer	Sequence ‘3-‘5
V258L_P259I AldO$_{Tf}$ Fw	CATCCTCTGATTGGAATGCCTGCAGTG
V258L_P259I AldO$_{Tf}$ Rv	CAGGCATTCCAATCAGAGGATGGCGAGGACC

Table S3. **Observed k_{obs} of the AldOs using deuterated glycerol as substrate**

enzyme	glycerol k_{obs} (s^{-1})	glycerol-d_8 k_{obs} (s^{-1})	ratio
AldO$_{Ab}$	1.46	0.36	4.1
AldO$_{Ch}$	1.34	0.50	2.7
AldO$_{St}$	1.32	0.45	2.9
AldO$_{Tf}$	1.49	0.32	4.7

Measured with the oxygen consumption assay at 25 ºC with 1 µM enzyme in 50 mM KP_i, pH 7.5 and 250 mM of glycerol or glycerol-d_8 (all hydrogens are replaced by deuterium).

***Table S4.* Single and double mutants performed on AldO$_{Tf}$.** Each X represents one of the double mutants and the header of the first column represents the single mutants. Marked in red are the single and double mutants that showed improved PELE profiles.

	P259N	P259A	P259L	P259I	P259V	P259T	P259R
V258L	X	X	X	X	X	X	X
V258M	X	X	X	X	X	X	X
V258I	X	X	X	X	X	X	X

	P262D	P262S	P262A
M261Q	X	X	X
M261V	X	X	X
M261L	X	X	X
M261I	X	X	X
